# Embracing Metagenomic Complexity with a Genome-Free Approach

**DOI:** 10.1128/mSystems.00816-21

**Published:** 2021-08-17

**Authors:** Izaak Coleman, Tal Korem

**Affiliations:** a Program for Mathematical Genomics, Department of Systems Biology, Columbia University Irving Medical Center, New York, New York, USA; b Department of Obstetrics and Gynecology, Columbia University Irving Medical Center, New York, New York, USA; c CIFAR Azrieli Global Scholars program, CIFAR, Toronto, Canada

**Keywords:** assembly, genomics, metagenomics, microbiome

## Abstract

A central paradigm in microbiome data analysis, which we term the genome-centric paradigm, is that a linear (non-branching) DNA sequence is the ideal representation of a microbial genome. This representation is natural, as microbes indeed have non-branching genomes. Tremendous discoveries in microbiology were made under this paradigm, but is it always optimal for microbiome research? In this Commentary, we claim that the realization of this paradigm in metagenomic assembly, a fundamental step in the “metagenomics analysis pipeline,” suboptimally models the extensive genomic variability present in the microbiome. We outline our efforts to address these issues with a “genome-free” approach that eschews linear genomic representations in favor of a pan-metagenomic graph.

## COMMENTARY

Microbiomes often contain hundreds of species, with a highly complex metagenomic structure; even distantly related microbes share genomic material (1, 2) due to vertical inheritance and horizontal transfers, and even closely related strains diverge (2–4). Variation is present within and between microbiomes (2–5), and occurs over relatively short timescales (4, 6). Understanding this variability is critical for topics such as emergence and maintenance of antibiotic resistance (7), in which horizontal gene transfer plays an important role (8). It has also been associated with host phenotypes (2–6), pinpointing specific genomic regions that are potentially adaptive to a particular host. In a recent study, we showed that a functional analysis of variable regions can even offer mechanistic hypotheses explaining such associations (2).

**FIG 1:**
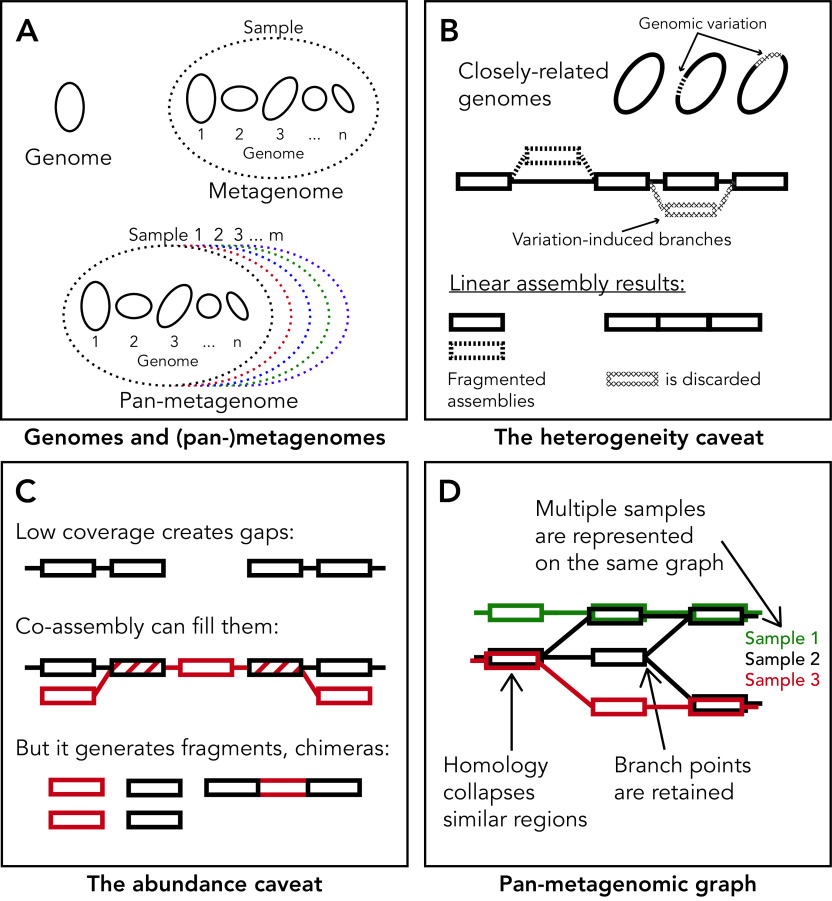
(A) Visual comparison between a genome; a metagenome, the collection of all genomes from a sampled microbial community; and a pan-metagenome, a collection of genomes, each deriving from one of multiple sampled communities. (B) The heterogeneity caveat: genomic variation between closely-related genomes (dashed sections) induces branching structures in assembly graphs (dashed nodes and edges). Linear assembly breaks down these structures, resulting in either fragmented contigs or the removal of variable regions. (C) The abundance caveat: undersampling of low-abundance genomes creates gaps in their assemblies. Co-assembly attempts to exploit information from close-matching genomes in other samples (red path) to fill these gaps. Some regions from these genomes are identical (diagonally striped nodes) and facilitate co-assembly; others are divergent, and introduce additional branching to the graph. This may result in either chimeras or fragmented contigs, and lower-quality assemblies in general. (D) We propose a graph-based representation of the pan-metagenome that addresses the caveats of the current paradigm. Our representation models metagenomic data across multiple samples, while keeping track of the originating sample of each sequence (red, black, and green). Sequence homology is used to collapse similar genomic regions (overlapping nodes), attenuating excessive branching within the graph in order to reveal variation at different scales with no information loss.

Variable genomic regions are likely poorly represented in reference genomes. Reference genomes are assembled from different populations, clinical conditions, or habitats, and have therefore been exposed to different environments and selective pressures. This means that they, and the variable genomic regions they encode, are likely irrelevant to the samples under study. A major promise therefore lies in *de novo* assembly, which directly models all the information present in a metagenomic sample. Recent studies, however, have demonstrated that state-of-the-art assemblers work well mostly for highly abundant strains with low heterogeneity (9), and are depleted of critical components such as mobile genetic elements (10). Here, we claim this is a direct result of the genome-centric paradigm. We argue for a “genome-free” approach, which does not attempt to produce linear assemblies but instead uses a “pan-metagenomic” graph (Fig. 1) that directly represents genomic variability across microbes in multiple samples. While we focus on analysis of short-read sequencing data, similar arguments could be made for long-read data. Our belief is that this approach offers a better framework for studying genomic variability in the microbiome.

## THE GENOME-CENTRIC PARADIGM FAILS TO CAPTURE THE PAN-METAGENOME

Contemporary assemblers (11–14) follow a similar process that realizes the genome-centric paradigm: sequencing reads are tiled into an assembly graph, which is then traversed to find paths representing linear contigs supported by the data. The goal of these assemblers is to generate the longest linear contigs possible, as reflected in some of the metrics used to assess assembly quality, such as N50.

Generating linear contigs is done at the cost of disregarding variation. When an assembler reaches a variation-induced branching structure in the graph (Fig. 1B), either one branch is selected over the other using some heuristic, such as removal of low-abundance variants that are assumed to originate in sequencing errors, or the branching structure is broken into multiple non-branching contigs (15, 16). In either case, the information contained in branched structures, which directly represents variability, is lost for downstream analyses. Indeed, assemblies of heterogeneous strains are typically poor in quality (9), likely due to sequence heterogeneity creating complex branching topology that assemblers cannot resolve, and instead fragment. This heterogeneity caveat of disregarding variation has a major impact on mobile elements and horizontally transferred genes, which are typically depleted from assemblies (Fig. 1B) (10).

Albeit less directly, the genome-centric paradigm also affects the assembly of low-abundance strains. A recent large-scale study demonstrated that high-quality metagenome-assembled genomes are generated only for genomes with approximately 10 to 20× coverage, attainable only for the most abundant strains in each sample (9). It is likely that strains with lower abundance simply lack the coverage that will facilitate a high-quality assembly from a single sample. This issue could be addressed by using information from closely-related strains present in other samples, an approach termed “co-assembly.” Co-assembly, however, also introduces additional complexity to the assembly graph, generating branches representing heterogeneity and homology between similar strains from different samples. As with the heterogeneity caveat, assemblers typically break these branches, resulting in fragmented contigs. In some cases, they might even traverse paths through them, introducing chimeras—contigs composed of multiple different strains. Consequently, co-assembly under the genome-centric paradigm reduces the quality of assemblies (17) and is not commonly used in our field (9, 18, 19). We refer to this effect of the genome-centric paradigm on assemblers as the abundance caveat (Fig. 1C).

In summary, the realization of the genome-centric paradigm in metagenomic assembly results in a suboptimal representation of the variability across microbiomes, particularly evident in low-abundance and heterogenous strains. At the heart of both the abundance and heterogeneity caveats is the fact that to comply with the genome-centric paradigm, and generate linear contigs, assemblers need to resolve branching structures. These structures, however, directly encode the genomic variability that we are interested in. It is not a surprise, then, that some reference-based methods attempt to detect exactly these branching structures by analyzing clipped read-mappings or variations in read-coverage (2, 20). We propose a more direct approach.

## BEYOND GENOMES: MODELING THE PAN-METAGENOME

In order to model variability within and across microbiomes, we are shifting our analytic representation of metagenomic data away from the genome-centric paradigm, toward the non-linear graph-based representation of the pan-metagenome: the entire collection of genetic elements present across multiple metagenomes (Fig. 1A). We use this representation to better model genomic variability in the microbiome, retaining the non-linear branching structures that encode variability. Being pan-metagenomic, our graph jointly models data from multiple samples. The originating samples of each sequence are recorded, facilitating comparative analyses. As we detail below, our framework also addresses the heterogeneity and abundance caveats (Fig. 1D), and could form the basis for extensive downstream analyses.

Heterogeneity induces a complex and nested branching structure in the pan-metagenome. Single nucleotide polymorphisms (SNPs) and small indels occur within larger structural variants, which may themselves show internal repetitive structure or homology to other genomes. In an attempt to construct long, linear contigs, assemblers resolve these branching structures; the consequential loss of variant information is the heterogeneity caveat. While we want to retain these branching structures and the information they encode, we also wish to control and attenuate the complexity of the resulting topology, in order to facilitate downstream analyses. We therefore use sequence homology to determine when branching should occur: sequences that are homologous according to a user-defined threshold are joined together, providing control over the topological complexity of the graph, without falling to the heterogeneity caveat. A similar approach was recently applied to long-read assembly (21). Our ability to simplify topology allows us to reveal the large-scale structural architecture of the pan-metagenome without losing fine-scale variation.

By combining information on closely-related strains across samples, co-assembly could improve the genomic information modeled for each strain. At the same time, it introduces additional complexity in the form of branched structures. As described for the abundance caveat, current assemblers either break down these structures or traverse chimeric paths through them. We approach this problem differently. Whereas current co-assembly approaches operate “blindly,” without utilizing information about the originating sample of each read, we use “informed co-assembly,” which exploits both this information and information about the genome sequence recoverable from each sample. This allows us to intentionally introduce chimeras when we believe that, based on recoverable sequence, two strains from different samples are similar enough such that a gap in one can be filled with sequence from the other. At the same time, we are able to ignore branching structures representing homology between distant strains, as if assembly within these regions was performed in a sample-specific manner. Consequently, informed co-assembly within our framework mitigates the adverse properties of co-assembly under the genome-centric paradigm. We flag the chimeras we introduce, enabling flexible and informed use of chimeras by downstream analyses.

Embracing non-linearity facilitates downstream applications that analyze variability: First, sample-specific content that is missing from reference genomes is available for comparative analysis. Second, known topological features, such as those induced by structural variations or lateral gene transfers, can be directly identified in the graph and associated with various phenotypes (e.g., host disease) by examining the originating samples of each sequence. Finally, new and complex topological features of importance can be identified directly by examining the topology of the graph in light of such associations with phenotypes. Beyond the study of variation, we posit that almost every analysis could be performed and potentially improved by considering the pan-metagenomic graph. For example, by applying binning algorithms (22, 23), sequences in the graph can be assigned to their harboring microbes and taxonomically classified. Additionally, the graph itself can be used as a reference, using read-to-graph mapping methods (24). Finally, sequence coverage per sample can be used to calculate gene and taxon abundance estimates; it has been shown that such estimates are improved by consideration of shared genomic elements (2, 25), which are comprehensively available from the topology of the graph.

Our vision is to use our framework as a bedrock for an unbiased and systematic study of the pan-metagenome and its interactions with the host. We are developing methods that directly analyze this pan-metagenomic graph, doing away with the prevailing separation between the assembly and analysis stages. These methods have access to complete information about variability and the genomic topology encoded in our graph, which is typically unavailable with current analysis pipelines. In the coming years, we hope to leave behind the genome-centric model, and instead use high-resolution analyses of the pan-metagenome to accelerate our understanding of how genomic variability shapes the relation between the host and the microbiome.
